# Functional Diseases

**Published:** 1908-03-28

**Authors:** 


### The Hospital

A Journal of

The Medical Sciences and Hospital Administration.

New Series. No. 54, Vol. II. [No. 1126, Vol. XLIII.] SATURDAY, MARCH 28, 1908.

### FUNCTIONAL DISEASES.

The chapter of medical history which deals with functional diseases is not by any means a grateful one to read. The dark ages before the days of Harvey we may fittingly neglect, for the whole of medicine seems to have been buried to an equal depth in theories and superstitions. But while in the last three hundred years our knowledge of organic diseases has advanced with almost marvellous strides, the record of the functional group remained until quite recently a dismal story of magic, charlatanism, and, it is to be feared, professional cowardice. It is a significant fact that the two men most prominently associated with the treatment of functional diseases in the eighteenth century have gone down to history as impostors of the worst description. These were Mesmer and Cagliostro. Mesmer was a qualified physician, who, dabbling in astrology—as was the custom of his time—evolved the theory that the influence of the stars upon the body could be made manifest by the application of magnets. At a later date he discarded magnets, and, crediting himself with some occult transmissible force, obtained an immense vogue among the neurotic element of Paris.

Mesmer's practices, no doubt, were those of a charlatan. But the facts of his power remained, and were so obtrusive that a Government Commission sat to consider them. Its report admitted most of the facts, which were indisputable enough, but denied the existence of animal magnetism as the agent of their production. Very presently the whole business fell into disrepute and an oblivion which Mesmer, but not his subject, emphatically deserved. This disrepute was deepened by the practices of that king of quacks, Cagliostro. It is true that he did not concern himself much with diseases, except towards the close of his extraordinary career, being far too deeply engrossed with the restoration of youth and other paying employments; but his trafficking with what we now know as the phenomena of suggestion was sufficient to seal the disapproval of the whole subject already aroused by mystic séances of Mesmer.

The first serious attempt to grapple with the problem of mental influence in relation to bodily function was made by James Braid, of Manchester, in 1841. Braid, attending a mesmeric séance in a spirit of pure scepticism, was surprised to find an apparent reality in the phenomena of the mesmeric trance. Curiosity being excited, he made some experiments himself, and succeeded in inducing a somnambulistic condition by fixing the gaze of his subject upon the mouth of a bottle held above, but close to, the eyes. By this experiment he demonstrated the cardinal fact that the manifestations of mesmerism depended not upon a mysterious effluence from the experimenter, as Mesmer taught, but upon a physiological condition of the subject. This was, of course, an immense stride, for it dragged the subject from the slough of mysticism, which had so paralysed the orthodox profession in their dealings with it. None the less, in spite of Braid's sound and honest work, the evil associations of the past continued to exercise their baneful influence. The profession still held aloof, although it was proved to demonstration that both mesmerism and hypnotism (as Braid renamed the influence known under Mesmer as animal magnetism), whatever their cause and explanation, were physiological facts. It is for this aloofness that the medical profession deserves the charge of cowardice. It may be argued that its anxiety to avoid contamination by contact with a subject which was certainly well pitched by its previous patrons was a sign of grace and a testimonial to professional integrity. But, on the other hand, science demands an unswerving service from her devotees. Braid had shown that the hypnotic state was a physiological condition, and no excuse can properly be admitted for the neglect exhibited by the profession in following the matter up. For thirty years from the time of Braid hypnotism lay dormant. In 1887 the celebrated Charcot was appointed to inquire into a new method of treatment by metallic discs. He, like Braid, found the phenomena genuine, and for some time took up the treatment of functional diseases by hypnotic suggestion with great enthusiasm. The reputation of Charcot gave a great stimulus to the study of the subject. Liébeault, of Nancy, and Heidenhain, in Germany, both devoted much attention to it, but the views of the three schools as to the explanation of it were antagonistic. Charcot and the Paris school regarded the hypnotic state as a morbid phenomenon, allied to hysteria. The Nancy school considered it as a mere product of suggestion, while Heidenhain attributed it to an inhibition of the higher cerebral centres by rhythmical stimulation of the senses. For some time the vogue persisted, but once again fate was unkind. It began to be muttered that in the schools where hypnotism was practised hysterical manifestations were encountered of a degree not to be matched elsewhere; and, indeed, there seems no doubt that the suggestibility of the clientèle was enhanced by repeated experiments. Charcot himself abandoned the practice, which thus fell once more into the hands of the travelling charlatan.

But of late a saner spirit has come upon the scene of functional disease. Hypnotism, if not dead, is moribund in therapeutics, for it has been shown that its phenomena can be obtained by mere suggestion in the waking state. Reflection and the weight of daily experience have to this extent justified the school of Nancy, in that they have proved suggestibility to be no morbid phenomenon but a quality exhibited in varying degrees by all normal individuals. The power of ideas in producing and dissipating functional troubles is being more and more realised, and thus the treatment of such maladies is being attacked by the psychical route already long trodden deviously by the patent-medicine vendor and mesmerist. At the present time the psychical treatment of functional disease is twofold. One school advocates indirect suggestion, the other a direct appeal to the reason. The method of the latter school is founded upon the premise that the permanence of most functional diseases is due to the false conclusion that every disturbance of function postulates an organic cause, and that the symptomatology in any given case is dictated by harmful auto-suggestions and fears. The aim of the treatment—called by Déjerine "persuasion," and by Dubois "rational psychotherapy"—is to explain frankly to the patient his morbid suggestibility, to illustrate the faulty conclusions to which he has been a victim, and by educating his critical faculty to give him a lasting weapon of defence. It is clear that, if successful, this method is to be preferred to the other, which merely replaces a harmful suggestion by a beneficent one, leaving the patient as susceptible as ever to the influence of his uncriticised ideas. In this matter we still lag behind our colleagues on the Continent, but our eyes are beginning to be opened, and one may hope that the later history of functional diseases will be more creditable to medicine than the earlier chapters.

